# Features and Practicability of the Next-Generation Sensors and Monitors for Exposure Assessment to Airborne Pollutants: A Systematic Review

**DOI:** 10.3390/s21134513

**Published:** 2021-06-30

**Authors:** Giacomo Fanti, Francesca Borghi, Andrea Spinazzè, Sabrina Rovelli, Davide Campagnolo, Marta Keller, Andrea Cattaneo, Emanuele Cauda, Domenico Maria Cavallo

**Affiliations:** 1 Department of Science and High Technology, University of Insubria, 22100 Como, Italy; andrea.spinazze@uninsubria.it (A.S.); sabrina.rovelli@uninsubria.it (S.R.); davide.campagnolo@uninsubria.it (D.C.); mkeller@uninsubria.it (M.K.); andrea.cattaneo@uninsubria.it (A.C.); domenico.cavallo@uninsubria.it (D.M.C.); 2 Center for Direct Reading and Sensor Technologies, National Institute for Occupational Safety and Health, Pittsburgh, PA 15236, USA; cuu5@cdc.gov; 3 Centers for Disease Control and Prevention, Pittsburgh, PA 15236, USA

**Keywords:** low-cost sensors, citizen science, miniaturized monitors, exposome, mobile app, wearable monitors

## Abstract

In the last years, the issue of exposure assessment of airborne pollutants has been on the rise, both in the environmental and occupational fields. Increasingly severe national and international air quality standards, indoor air guidance values, and exposure limit values have been developed to protect the health of the general population and workers; this issue required a significant and continuous improvement in monitoring technologies to allow the execution of proper exposure assessment studies. One of the most interesting aspects in this field is the development of the “next-generation” of airborne pollutants monitors and sensors (NGMS). The principal aim of this review is to analyze and characterize the state of the art and of NGMS and their practical applications in exposure assessment studies. A systematic review of the literature was performed analyzing outcomes from three different databases (Scopus, PubMed, Isi Web of Knowledge); a total of 67 scientific papers were analyzed. The reviewing process was conducting systematically with the aim to extrapolate information about the specifications, technologies, and applicability of NGMSs in both environmental and occupational exposure assessment. The principal results of this review show that the use of NGMSs is becoming increasingly common in the scientific community for both environmental and occupational exposure assessment. The available studies outlined that NGMSs cannot be used as reference instrumentation in air monitoring for regulatory purposes, but at the same time, they can be easily adapted to more specific applications, improving exposure assessment studies in terms of spatiotemporal resolution, wearability, and adaptability to different types of projects and applications. Nevertheless, improvements needed to further enhance NGMSs performances and allow their wider use in the field of exposure assessment are also discussed.

## 1. Introduction

### 1.1. Glossary and Terminology

To better explain the meaning of the terminology used in this review, the authors would like to clarify the definition of some terms that should not be taken for granted. This is needed to avoid misunderstandings while reading the following text. In the context of this review, a “sensor” is a component of a measuring instrument; more specifically, it is the sensing component that allows the performance of the measurement of the airborne concentration of pollutants, typically by relating a chemical–physical property of an airborne pollutant with a signal that can be detected by the instrument, related to the concentration of the pollutant, and then made available and interpreted by the evaluator. A “monitor” (also referred to as a “sensor system”) is an integrated device, i.e., a measuring instrument for pollutants airborne concentrations, which is equipped with one (single-parameter monitor) or more (multi-parameter monitor) measuring sensors, as well as with sub-components and other supporting components (e.g., battery, case, GPS (global position system) module, display, Bluetooth module) needed to create a fully functional and autonomous detection system. A sensor system can also include components that reside remotely from the physical sensor and include remote data transfer and data processing steps [1]. The goal of this review is to characterize what the authors intend to call “next generation” monitors and sensors (NGMS): this term refers to “miniaturized” and/or “low-cost” and/or “wearable” sensors and/or monitors. Concerning the definition of “miniaturized monitors” (MMs), in this study, the authors refer to a previous study [2] which identified MMs as those devices with a greater dimension smaller than 20 cm. The proposed dimensional criterion is not always strictly applied in the scientific literature, but it was an arbitrary subdivision with a certain level of subjective decisions. In any case, MMs are those devices having significantly lower dimensions than reference-grade instrumentation. Among MMs, a particular category of sensors and monitors are wearable monitors (WMs), i.e., small, lightweight monitors being used as wearables to provide real-time personal exposure measurements [3,4]. There is still a lack of a universally agreed upon definition of low-cost monitors (LCMs) [5]. Although some different definitions are available [6,7], the scientific community generally defines them as those having significantly lower costs than reference-grade instrumentation [4], in such a way that the acquisition of a single unit has a minimal impact on the budget for monitoring activities. For the purpose of this study, an LCM is defined as a monitor, the cost of which does not exceed the order of magnitude of a few hundreds of dollars [7,8].

### 1.2. Background

As well reported in the literature, air pollution is associated with several acute and chronic effects on the human health (e.g., cardiopulmonary disease, respiratory infections, diabetes, and lung cancer), the nature of which varies depending on the pollutants taken into consideration [9]. According to the World Health Organization (WHO), the majority of people spending their lives in cities are exposed to pollutant air quality levels that exceed the WHO limits [10]. Air quality monitoring networks are typically implemented at a regional or national scale, to verify compliance with air quality objectives and standards (e.g., those reported in Directive 2008/50/EC [11]). These networks consist of fixed monitoring stations located throughout the territory (in urban and suburban areas, typically). These fixed-site stations are, as a rule, equipped with reference-grade instrumentation, and can thus provide accurate and precise data for a general-purpose (i.e., verifying the compliance with regulations), but cannot accurately describe the variability of single citizens’ exposure to airborne pollutants with a high spatial and temporal resolution [12]. To have a complete and representative health impact assessment, the human exposure to airborne pollutants should ideally be evaluated overall, following the exposome concept defined in Wild et al., 2005 [13] and more recently well explained in Jiang et al., 2018 [14] (or rather, the exposure and determinants from conception onward, over a complete lifetime [15,16]). Improvement in exposure assessment methods could provide substantial advancement and increase the potential and the level of detail and depth of studies relating to air quality and health effects and epidemiological studies. For example, research studies linking personal exposure to airborne pollutants with subjects’ specific variables (e.g., performed activities, visited micro-environments, modes of transport, other individual behaviors and customs) are made possible only if it is possible to promptly collect the information (both exposure level and contextual information) of individual patterns [17,18]. These studies have already been conducted with portable monitors in the past few years, but the information collected may become even more comprehensive if the monitoring activity could be effectively extended to longer periods (weeks, months, years) and not limited to narrow time periods. From this perspective, NGMSs represent an important resource for implementing this type of study and promoting a “citizen science” approach, based on the active involvement of citizens, in this case by measuring their own personal exposure, aiming, for example, to find a link between exposure and its determinants and between health symptoms with their causes [19]. The typical characteristics of NGSMs (e.g., availability of real-time data, ease of use, low cost and small size, ease of data transmission and management, etc.) make them particularly suitable for citizen science applications. NGMSs can be used to retrieve measurement of air pollutants exposure for specific subjects (e.g., susceptible people, commuters) and can be used both indoors and outdoors [2]. In this way, citizen scientists will be able to investigate specific micro-environments (MEs) more deeply, better characterizing elements of subjective, spatial, and temporal variability, thus allowing a better characterization of exposure, sources of pollutants and managing risks even with more efficacy than what is currently possible. During the last years, the advancement in sensor technology and its miniaturization (e.g., portable and wearable sensors; sensors embedded in mobile phones) have also contributed to the spread of the exposome concept in exposure assessment studies [14]. Thanks also to these advances, the individual exposome could be evaluated more easily by completing life-time exposure data obtained by biological monitoring (internal doses) with easy-to-apply environmental monitoring approaches (external doses). Other than the availability of miniaturized measuring devices, further developments (e.g., the use of under-skin sensors or the automatization of data treatment processes) [20] can be recognized as useful improvements for exposome studies. For example, improvements in online data transmission could be useful for the creation of a wireless sensor network (WSN) [2]. Furthermore, the assessment of occupational exposures to chemicals must be performed in the framework of occupational risk management and in the context of workers’ health protection and disease prevention policy and regulation [21]. On this basis, occupational exposure assessment can be performed: (i) to check compliance with exposure limits; (ii) to assess personal exposure and exposure control efficiency and effectiveness; and (iii) for research studies [22]. These well-established issues are receiving increasingly more attention in recently introduced concepts such as “total worker health” [23] and “occupational exposome” [13], which have numerous points in common with respect to what has been said in the previous points and which can also benefit from the introduction of NGMS into the practice of occupational exposure assessment to chemicals.

### 1.3. Aim of the Study

The advancement in exposure monitoring practices should be driven by identifying and addressing specific needs and not by the availability on the market of the new technologies. Focusing on new solutions to solve the identified problems with the support of technologies is the only way to conduct scientific research effectively in this area. NGMSs represent a very promising technology to conduct and complement an exposure assessment study that, if developed and improved, can resolve the typical limitations that characterize traditional airborne pollutant monitoring methods. This systematic review is not about the entire “monitoring systems” but aims to understand which are the most used NGMSs reported in the literature, with the intent to catalog them, to facilitate their selection in future projects and monitor creation, and to advance their responsible adoption in future exposure assessment studies. In particular, this systematic review aims to focus on NGMS, i.e., a sensor or a monitor that is miniaturized and/or low-cost and/or wearable. All these types of NGMSs have been considered, not excluding one regardless because it did not meet one of the requirements.

In addition, by evaluating additional features and sensor characteristics (GPS, temperature (T) and relative humidity (RH) sensors, dedicated apps, battery packages, price, etc.), the review indicates the areas of interest that the scientific community of creators should improve in the next years, considering that there is definitely a temporal delay between the commercialization of new technologies and the publication of related scientific papers.

## 2. Materials and Methods

A systematic review of the scientific literature has been performed using the outcomes from three different databases (i.e., Scopus, ISI Web of Knowledge, and PubMed).

A list of keywords was arranged in queries (Table 1) following the writing rules required of ISI Web of Knowledge and Scopus.

**Figure 1:**
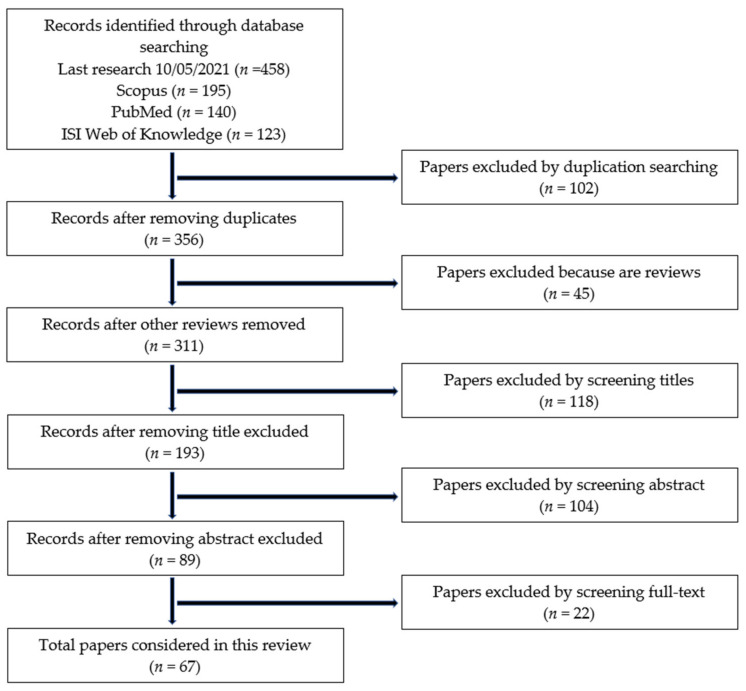
Flowchart of the papers which are the object of this review (modified from [24]).

For PubMed, it was decided to omit the keyword “pollut *” due to the large numbers of irrelevant results generated (*n* > 3640). The query structure was reorganized following the writing rules of each database. As reported in Figure 1, 195 papers were found in Scopus, 123 in ISI Web of Knowledge, and 140 in PubMed. The last research of the papers was completed on 10 May 2021 (weekly updates were performed until the date of submission of manuscript). After the (i) elimination of duplicates (*n* = 102), (ii) elimination of papers that were not original research articles (*n* = 45), and (iii) elimination of completely off-topic papers via title screening performed by two of the authors (G.F.; F.B.) (*n* = 118), the abstracts of the remaining papers (*n* = 193) were (iv) analyzed and those not relevant for the purpose of the study (*n* = 104) were excluded. Additional inclusion and exclusion criteria were then adopted to obtain the final list of papers investigated. First, only scientific papers written in English were considered. Since the review is focused on a newly developed topic, no time filters regarding the year of publication were used. Moreover, both mono- and multi-parametric monitors used in the paper under review were included in this work. Papers that were exclusively focused on exposure models were not considered. Only direct reading instruments and real-time NGMSs (as defined above) reflected the topic of this review, so all papers concerning substrate-based methodologies were discarded. The papers not reporting any information about the pollutants investigated using instruments and adopted sensors were not considered. From these steps, 89 papers were considered for the full-text reading step and 21 of these were further excluded. Finally, after the paper evaluation, following the inclusion/exclusion criteria mentioned above, 67 papers (Table S1) were reported and included in the present review.

## 3. Results and Discussion

In the last years, a rapid increase of interest around the development and the application of NGMSs was detected. This is testified by the fact that the scientific publications published on NGMSs are constantly growing. By applying the research queries described above, 34, 71 and 50 published articles were retrieved in Scopus, PubMed and ISI Web of Knowledge, respectively, when considering the period from 1975 (year when the first paper found applying the queries was published) to 2015. The number of published articles has grown significantly in recent years (2016–2021): 195 (Scopus), 140 (Pubmed) and 123 (ISI Web of Science) published papers were retrieved in this period. Technical features of the selected sensors are summarized in Tables S2 and S3 (Supplementary Materials), while other issues are discussed hereafter. All the articles under review were analyzed aiming to find the sensors used to measure the air concentrations of the airborne pollutants. In particular, if the sensor was included in a multipollutant monitor, it was cataloged as a single sensor. Other types of information were taken into consideration, such as the use of an integrated GPS system and the presence of T and RH sensors (Section 3.1). Due to their features, NGMSs are often able to directly communicate with specifically developed mobile applications (“mobile apps”). Therefore, mobile apps were also taken into consideration in this study. All the mobile apps that can communicate with monitors or sensors (mainly via Bluetooth technology) and are used to download and manage measurement data, without the necessity of a cable connection, were considered (Section 3.2). As we discovered, few papers have cited this type of technology, but it could be an important future development to help operators during the acquisition data phase. Applications of NGMSs in the field of environmental exposure assessment are presented hereafter (Section 3.3), without neglecting the improvement in citizen science that can arise due to the use of NGMSs (Section 3.3.1). Another important topic of this review regards the use of NMGSs in occupational exposure assessment studies (Section 3.4) and consequently the crucial role of the implementation of these technologies in the occupational hygiene field. To develop all these aspects, it is important to distinguish the potential role of sensors and monitors in exposure assessment studies but also to obtain behavioral changes of the subjects that use it. For example, considering the reviewed studies, there was no evidence of studies aimed at conditioning the behavior of the subjects as a consequence of pollutant exposure levels. This opportunity could be implemented in the future (e.g., introducing acoustic or lighting signals, alarm, or notification to the monitored subject) to take action immediately when the concentration levels exceed a defined or proposed threshold exposure value, to avoid exposure conditions which can be considered risky. Finally, to summarize, the weaknesses and strengths of this study have been analyzed (Section 3.5).

### 3.1. Sensors for Selected Pollutants

In 2008, the WHO recommended and targeted values for main air pollutants [25,26]. Among those proposed, this review focused on the following pollutants: nitrogen dioxide (NO_2_), ozone (O_3_), carbon monoxide (CO), volatile organic compounds (VOCs) and airborne particulate matter (PM) with an aerodynamic diameter below 2.5 µm (PM_2.5_) and below 10 µm (PM_10_) (the coding “PM” was applied to categorize NGMSs that can simultaneously analyze more than one fraction of particulate matter) (Table 2). After the full-text reading step, it was outlined that some pollutants were poorly investigated and the available evidence did not allow for an extensive discussion: for this reason, NGMSs for NO [25,27,28,29,30,31], NO_x_ [32,33], CO_2_ [31,34,35,36], SO_2_ [37,38], and BC [39] were not discussed in this review. As a first result, we found that the most commonly used sensors to monitor the selected air pollutant gases are those produced by Alphasense (www.alphasense.com; accessed on 22 April 2021; Great Notley, Essex, UK). These sensors are categorized as electrochemical (EC) sensors, based on an amperometric principle of operation for the quantification of nitrogen dioxide (NO_2_), ozone (O_3_) and carbon monoxide (CO) [29]. Furthermore, concerning the monitoring of VOCs, what emerged from the literature is that the most common instruments used for this scope are produced by Sensirion (SGP30 and SGPC3) [19,40]. Regarding the measurement of different-sized fractioned PM particles, the most used sensors are those produced by Plantower (PMS3003; PMS5003) and Sharp Electronics (GP2Y1010AU0F) probably also due to their low dimensions and costs. These sensors are based on physical light scattering (LS) processes. Due to the interaction of a light beam with PM, the beam is diffused partially and randomly in all the directions of space. The detection of the intensity and wavelength of scattered light contains information about particle size and/or volume [41]. In these NGMSs, the incident light source is usually a laser and light detection devices (photodiodes) are placed at specific angles to the incident direction. Temperature (T) and relative humidity (RH) sensors were also considered because NGMSs performance may vary significantly with the variation of these factors [37,42,43,44]. For example, when the RH is high, condensed particles and fog are detected and reported by particle monitoring instruments that do not have drying systems at the sample inlets, thus interfering with the measurement performance. This effect should be considered when using low-cost sensors [45,46,47] at the same as it was considered in past studies using time-resolved monitors [48,49,50]. Temperature is a key factor that has an impact on the reaction rate and gas vapor pressure. It could be observed that the QTF (quartz tuning fork) gas sensors’ (mass sensitive piezoelectric resonators) sensitivity decreases with increasing environmental temperatures. Therefore, the temperature-dependent sensitivity behavior needs to be accounted for in the QTF sensors calibration protocol to consider different real free-living environmental scenarios [51]. Regarding the sensors used to acquire T and RH data, there is no evidence that one sensor is preferred and/or more used than others, but the most selected brand is Sensirion. The investigated T and RH sensors are all based on the principle of capacitive sensing (CS) to measure RH values and on silicon band gap (SBG) semiconductors to measure T values. Finally, regarding the acquisition data about GPS information, very poor information were found: only 2 of 67 papers explain which sensor models are used in the respective studies (G.TOP FGPMMOPA6H [27] and Adafruit Ultimate GPS chip [52]). This is probably due to the fact that GPS sensors have a high energy consumption so it is preferred to use mobile phone-integrated GPS modules to save battery consumption (e.g., [53]).

### 3.2. Mobile Apps

A crucial role to improve the user interaction with the devices is played by mobile apps specifically developed for some of the NGMSs. In the last few years, this aspect has played an increasing role, especially as regards the storage and transfer of measurement data. The most important role in this sense has been played by technologies that allow the cableless (wireless personal area network) transfer of measurement data from the device (where they are temporarily stored in special data-loggers or memory slots) to the mobile app platform, where they can be viewed, processed, managed, and shared, if necessary. As said in Borghi et al. (2017) [2], the way to communicate and share scientific data is changing and some aspects are particularly interesting such as (i) communication and data transfer via wireless and (ii) data communication via web or smartphone applications. This generally saves time and is more practical than more laborious methods that require manual data download and subsequent processing. The most widely used method is Bluetooth technology, which is further improved with the development of Bluetooth low-energy technology (BLE) [78]. It allows easy and stable communication between NGSMs and a smartphone in which the mobile app is supported. In this review, 23 articles [19,27,32,35,37,38,39,50,51,56,60,67,69,77,79,80,81,82,83,84,85,86] out of 67 reports information about the use of any mobile app supporting NGMSs; most of those (13 apps) were developed on the Android platform [6,35,37,38,40,50,53,60,81,84,86,87,88], only one was developed on the iOS platform [81], and the remaining were not specified. As reported by Kanjo et al. [89], using a mobile phone to collect data can bring many advantages, especially related to the fact that (i) a large percentage of the population carries around mobile phones; (ii) many kinds of data can be processed, stored, and transferred easily by mobile phones; (iii) the collection of data should be more power-efficient because the acquired information are sent directly to the mobile phone. Due to these advantages, the use of mobile apps is considered one of the aspects that is sensibly improving exposure assessment studies, shortening and filling the distance between citizens and researchers. Different kinds of outputs are returned by the smartphone application, such as the concentrations of the investigated pollutants, date, time, and position; these outputs are generally reported in a user-friendly interface. All of these data can be plotted in real time on a graphical interface that allows users to immediately share important information such as exposition peaks, mean concentrations, limit values (e.g., AirCasting app by HabitatMap Inc. [50]). In future developments, to describing sensors and apps, it will be recommended to also investigate communication transmission technologies and common platforms/websites applied to these low-cost sensors, such as 4G, 5G, or Wi-Fi. For the platform, for example, the Edimax Airbox (https://airbox.edimaxcloud.com/ (accessed on 22 April 2021)) and LASS location-aware sensing system in Taiwan are used.

### 3.3. Applications in Environmental Monitoring and Exposure Assessment

As already discussed, NGMSs cannot totally replace traditional approaches in environmental exposure assessment regarding data reliability, but they can fill other gaps, such as improving data in terms of spatial and temporal resolution. However, although reliable measurements through reference instruments are (and will remain) fundamental, other features of NGMSs may outweigh some of their drawbacks, including lower measurement reliability. Traditional measurement methods require bulky instrumentation. Instead, thanks to their low weight and dimensions, NGMSs are generally miniaturized and/or wearable, which can minimize the interference on subjects’ normal activities. For all these reasons, innovative studies for environmental exposure assessment will probably need to exploit both traditional methods and NGMSs, or a combination of them, to allow the investigation of a wide range of different scenarios and subjects’ categories or populations [49]. A range of low-cost air quality sensors are now available on the market, thanks to the fast-growing field of sensing technology. Most of these monitors provide quantitative information of pollutant concentrations, in addition to being generally quite easy to use [33,77]. The performance of these low-cost miniaturized sensors must be evaluated, especially in-field. Moreover, their comparability (compared to reference methods [90]) should be carefully evaluated. Using these miniaturized sensors as a support to fixed air quality monitoring networks, both in indoor and outdoor environments [49], it should be possible to obtain a more representative characterization of the subject’s exposure and achieve a wider spatial coverage. With the continuous improvement of these technologies, it could be possible to develop and use ubiquitous networks of NGMSs, by different subjects and entities (i.e., governments, municipalities, or individuals). Furthermore, many end-user applications shall be available. These applications can be installed and used by anyone, not only by experts in air pollution monitoring, who can also select the right type of NGMSs for the right purpose and to obtain the data needed. Nevertheless, the data interpretation by non-experts could introduce issues that may affect the validity of the results [6]. This concept refers to the already introduced citizen science approach, defined as scientific research conducted, in whole or in part, by amateur (or non-professional) scientists. The application of these technologies is set to grow and the conversations with communities are expanded by the current low-cost sensing technologies, which also supplement the routine ambient air monitoring networks [6]. Through the use of machine learning, Chew et al. (2019) [53] have been able to demonstrate that by using monitors for the evaluation of personal pollutant exposure, equipped with accelerometers, it is possible to identify periods of biking through the subjects day. Since personal exposure data is related to the respiration rate [53], thanks to the finding mentioned above, the estimation of the dose of potential pollutants inhaled has become possible applying the use of NGMs in exposure assessment studies. Sinaga et al. (2020) [72] outlined that, thanks to the advent of NGMSs, nowadays it is easier to investigate the daily exposure of citizens that live in developing countries, even if they usually do not have many resources to perform these evaluations. In their study, the most contributive factors of PM exposure were identified as mosquito coil burning and factory smoke and it has been taken as reference information to formulate policies and guidelines that aim to reduce citizen exposure and improve health protection [72]. Obtaining expensive instrumentation to monitor air quality is not always foregone, especially in developing or industrializing areas, but NGMSs can solve this problem due to their low cost and easy applicability [76]. Win-Shwe et al. (2020) [91] indicated that continuous assessment of personal exposure level is possible using the NGMS developed in their study, also matching NGMS with mobile sensing technologies. The authors are planning to give health education to the public regarding lifestyle in microenvironments with the scope to reduce indoor air pollution [91]. Barkjohn et al. (2020) [73], using several NGMSs, have pointed out that reducing the infiltration of outdoor air in homes and decreasing pollution at the city or country level can reduce the personal exposure of citizens. The project conducted by Chen et al. (2020) [57] investigated the personal exposure of students to PM_2.5_ wearing NGMSs during school hours in a two-month campaign. The personal exposure of the students can be influenced by outdoor pollution, caused by nearby sources, and it must be evaluated also monitoring air quality outside the school building. The monitoring campaign outcomes showed that short-term and acute events (e.g., resuspension of particles due to students’ movements) are more significant in terms of contributing to exposure than outdoor air pollution. The suspended particle characteristics significatively influence the exposure of the subjects due to their high inhomogeneity, which contributes to increment its variability [57].

Overall, one of the biggest problems of NGMS technologies regards the power supply. Most of the devices and sensors available on the market are supplied with ion lithium batteries [19,40,60,73,76]; unfortunately, they cannot run for many hours without recharging. Despite this, the scientific community is continuously investing resources to improve the autonomy and reliability of these device’s batteries because whilst some devices are able to work for 24 h and more, most show the abovementioned limit. If NGMSs will soon be able to be functional over medium to long periods (i.e., more than 24 h), it could represent an enormous step forward in the field of environmental exposure assessment studies, since subjects’ exposure assessment will be easily extended over the entire day (or even longer periods, e.g., weeks, months); thus, very representative exposure profiles will be technically achievable and accessible.

#### 3.3.1. Improvement in Citizen Sciences

One of the reasons encouraging scientists to continuously develop NGMSs is the fact that these devices can be used to investigate air quality together with citizens to support well-informed actions and communicate the problems regarding this topic to the general population, raising the attention of politicians. Few studies underline the importance of citizen science in the field of air quality to evaluate the impact of everyday citizen life and daily routine [32,50,56,60,92]. In 2018, the ECSA (European Citizen Science Association) [93] created a collaboration between scientists and industries with the aim to encourage networking in the field of citizen science, aiming to reach a constant fueling of resources, not only economic but also in terms of ideas, research needs, and initiatives. Following the trend of NGMSs for air quality monitoring, several citizen science projects are developing. The application of NGMSs-specific features (i.e., GPS and mobile apps) in citizen science studies may stimulate interest in citizens. For example, GPS sensors allow citizens to track their daily movements, so that they can select the route with lower airborne pollutant concentrations [40,91]. Mobile apps allow citizens to interact with monitors and sensor fueling that sense participation in research projects, which is the base of citizen science studies [50,94]. All of these initiatives have stimulated discussions with policymakers and influenced political decisions [95]. Moreover, citizen science in environmental monitoring can increase the potential for acquiring new knowledge while creating information that goes into policy formulation, planning, and management activities at various levels of government [95].

### 3.4. Applications in Occupational Hygiene 

As reported above, most of the papers analyzed in this review showed that the use of NGMSs is widespread in environmental exposure and environmental health studies, some of which also directly and actively involved citizens in exposure measurements. NGMSs are used to support the reference-grade monitoring instruments and environmental health policy and strategies. To date, the use of NGMSs in occupational hygiene applications is less frequent, mainly because policy- and legislation-based decisions have the strictest performance requirements for precision, accuracy, completeness, and detection limit of data [95]. Nevertheless, NGMSs sensing devices can offer new opportunities in the field of occupational safety and health management [4,22,41,45,61,62,92,96,97,98,99]. Some of the most interesting applications of NGMSs are reported hereafter. NGMSs were applied in physically demanding and hazardous construction settings [97] with the aim to mitigate the high risks associated to these work tasks. Even though that is not the focus of this review, various smart bracelets, wristbands, and smartwatches incorporate numerous sensors that allow to track health and exercise and combine the capabilities of a smartphone with a wristwatch. The purpose is to exploit the capabilities of wearables to change the way workers interact with their environment and enable them to monitor critical, environmental, and physiological data and process it to gain situational awareness. Data acquired by conventional sampling becomes available weeks after sampling and wearables usually provide a single measurement of one hazard, typically integrated over a single work shift. In the last decades, industrial hygienists have been using direct reading instruments (DRIs) and real-time monitors for gas/vapor and PM monitoring. NGMSs also provide measurements that are immediately available for actions and interpretations providing continuous monitoring of several hazards throughout the workplace. NGMSs are still smaller, lighter, and more powerful and connected than the instrumentation of recent decades. The identification of several sources of hazards has been possible thanks to these measurements, which are also used to formulate strategies for improved control and continuously evaluate their effectiveness. A shift to comprehensive exposure assessment is possible thanks to this departure from the conventional sampling usually adopted until nowadays and the priority that workers are adequately protected from workplace hazards will undoubtedly be improved. Once matched with a position tracking system, in the future, these data will also be used to evaluate the personal exposure of a single worker and can be modeled while they move through the workplace [62]. The application of NGMSs may have several advantages for workers regarding workplace safety monitoring [100]. For example, integrating real-time data with machine-learning models, a subset of artificial intelligence that is concerned with creating systems that learn or improve their performance based on the data they use, can exponentially raise the probability of preventing and limiting the potential risks associated with the industrial environment [96]. Moreover, the development of newer software toolkits and microprocessor platforms is powering the WSN systems. A WSN is a network of several sensors that can communicate with each other and with a central controlling unit that collects all the information coming from all the devices. By modelling this information, it could be possible to create plant risk maps, and consequently manage the risk at each workplace, with the aim of improving the occupational health and safety system [61]. As suggested by Goede et al., 2020 [22], high-resolution data from real-time/direct-reading instrument sensors can be used to enrich estimates from models that predict exposure to chemicals in the workplace. By modeling the information acquired by the sensors, recalibrating, refining, and validating existing (time-integrated) models, scientists will be able to improve worker’s security and health in the workplace. New approaches such as “occupational dispersion models” (e.g., interpolation/computational fluid dynamic models, and assimilation techniques), paired with sensor data, will be specifically useful. Through early warning systems, source finding, and improved control design, these techniques may be used to develop site-specific personal exposure maps which could significantly support the aim to mitigate worker exposure [22]. It is also necessary to elaborate on the meaning of “exposure assessment” because it is not obvious that its intrinsic meaning could be directly applied in occupational hygiene applications when using NGMSs. For example, when NGMSs are not only used to monitor the workers’ exposure (i.e., for exposure assessment purposes), but also to conditionate the behavior of the workers (i.e., by providing real-time warning to the worker experiencing high exposure conditions and therefore suggesting a change in the performance of the job task to reduce the level of exposure). The result of this kind of application will not only be that of having a representative measure of the exposure of the worker in real conditions, but rather an “exposure-based real-time risk management” in which the behavior (and consequently the exposure) of the worker is modified in real-time, thus also providing a sort of exposure-driven risk management.

### 3.5. Overall Discussion

In summary, NGMSs could provide substantial benefits (including lower efforts at lower cost) when applied to the monitoring of exposure to airborne pollutants in both general environments (i.e., for general populations) and occupational settings (i.e., workers’ occupational exposure), if compared to traditional exposure assessment methods, which rely on sampling devices (i.e., by means of sampling pumps or diffusion methods), sampling substrates (e.g., sampling filters, adsorbent substrates), and on the subsequent analytical phase (e.g., gravimetric determinations, chemical characterizations). In more detail, one of the advantages of NGMSs is to provide new insights on exposure dynamics due to their ability to collect data at greater spatiotemporal resolutions (i.e., direct-reading methods) [76]. Furthermore, NGMSs can report and process the data as soon as they are collected and while the instrument is still deployed (i.e., real-time analysis). Therefore, due to their features (i.e., reduced cost, ease of deployment, direct reading capabilities together with the wireless network ability and the possibility of integrating them with other exposure estimation methods), new ways of collecting and sharing environmental and occupational exposure information has become possible using NGMSs [4,22]. For these reasons, both in environmental and occupational hygiene, not only is the need for accurate evaluation of human exposure to airborne pollutants confirmed and reiterated, but a step forward is required as regards the methods, techniques, and technologies to be used for this purpose.

#### 3.5.1. Issues and Considerations

Despite the advantages related to the use of NGMSs reported above, it is important to underline that these kinds of technologies should be deeply evaluated before use, especially in terms of measurement precision and accuracy [25,28,50,68]. Therefore, despite the expected advantages, the use of NGMSs for exposure assessment can also present drawbacks and difficulties [2,6]. NGMSs are generally less reliable (in terms of accuracy, sensitivity, precision, and specificity to the chemical/variable of interest) if compared to high-end devices [101,102,103]. Overall, NGMSs are being successfully used complimentary to reference monitoring [104], but they are not yet validated as alternative techniques for (or to replace) reference instruments (especially for purposes of mandatory monitoring) [103]. Although some studies are available [2,6,105], definitive and comprehensive evaluations concerning the agreement between sensor systems and reference instrumentation are not available, and neither are performance evaluations of NGMSs in different exposure scenarios. Furthermore, unlike what happens for reference-grade instrumentations that are subjected to comprehensive regulatory standards and processes for evaluation and certification, only a few standards exist for NGMSs [106]. Moreover, biases in the acquisition and interpretation of the data obtained with NGMSs can be derived from different sources of measurement error and interference, which arise once operating in the field and which cannot be completely covered in the development and calibration phases carried out in the laboratory [101]. For these reasons, NGSMs should be operated applying rigorous quality assurance and quality control protocols [4,49]. Focusing on the concept mentioned above, Fishbain et al. [107] proposed a sensor evaluation toolbox (SET) for evaluating air quality micro sensing units, based on different performance measures and environments. Included within the SET are new schemes for evaluating several sensors’ capabilities. Each of the evaluation criteria allows for assessing sensor performance, together constituting a holistic evaluation of the suitability and usability of the sensors in a wide range of applications. By applying this toolbox, the authors outlined that while specific sensors would be graded poorly using the traditional evaluation tools, these would be more than sufficient for many of the aforementioned applications, such as citizen science and exposure estimations. In summary, although accurate measurements are important for monitoring environmental and occupational exposure, the previously described advantages may outweigh some of these disadvantages [4], depending on the reason behind monitoring.

#### 3.5.2. Preliminary Outcomes

The principal results of this review show that the use of NGMSs is more and more common in the scientific community for both environmental and occupational exposure assessment to airborne pollutants. Despite the majority of studies also making use of traditional (reference-grade) instrumentation used as reference methods [32,33,42,52,76,82,106,108,109,110], NGMSs will possibly gradually replace, or at least complement, reference instruments in several applications. It is worth noting that some ethical issues could be raised due to privacy motivations because wearable sensors can acquire personal data concerning the health of the subjects investigated, as well as their habitudes, activities, and movements during the monitoring period. If the problem is quite simple to solve in the environmental exposure assessment studies by recruiting volunteers, the occupational hygiene application of wearable sensors may pose several issues. As mentioned, the major weakness related to the use of NGMSs refers to their performance and reliability, an issue well established and reported in several studies [32,71,111]. For this reason, NGMSs should also be paired with traditional sampling techniques to be able to correct the data acquired applying a correction factor obtained by comparing the two different methods [49]. Moreover, another issue in the use of NGMSs, as well as other portable instruments, refers to the autonomy of the batteries used to supply the monitors. They must be as powerful (and miniaturized) as possible to increment their autonomy (ensure their wearability); unfortunately, to the best of our knowledge, we are far from the goal of continuously monitoring many hours of the subject’s exposure without recharging the instruments. Nevertheless, the strengths (i.e., low costs, low weight and dimensions, high spatiotemporal resolution, user-friendliness) of these emerging technologies underline that the increasingly popular approaches will be easier to apply.

#### 3.5.3. Latest Developments

Further developing NGMSs and investing resources in this field will provide scientists with better-performing devices in terms of data accuracy, durability of batteries, low-weight, and dimensions. For example, NGMSs able to provide continuous and real-time data allow to detect exposure peaks in different scenarios; they can be worn by a subject without compromising daily routine and most of them can be operated by anyone, regardless of whether they have specific competencies in the field (e.g., citizens or workers). In this regard, it is good to remember that skills and expertise in exposure assessment must not be taken for granted; therefore, the design of these studies and the interpretation of the results should in any case be entrusted to experts in the field. Finally, NGMSs, thanks to their characteristics, can be used to obtain data in a wide range of applications (e.g., several microenvironments such as occupational fields; indoor or outdoor environments; houses; specific rooms; a specific moment of a day, such as commuting) and they can be used in various experimental designs. Other technical aspects that have emerged from this review refer to the durability of batteries, the use of mobile applications, and the versatility of NGMSs in different settings. In particular, the range of durability of the batteries used to power the sensors is not enough to conduct studies that continually monitor for long periods without recharging; this is the main reason that does not yet allow their use in exposome studies. Moreover, the usage of mobile applications to control the sensors and download data has highly improved in the last years but further implementation is necessary to automize this process as much as possible, aiming to increase data accessibility and usability. Finally, the reviewed articles underline the versatility of the NGMSs, which can be used to conduct several types of studies, principally thanks to their high spatio-temporal resolution and very low impact in the daily routine of investigated subjects.

### 3.6. Weakness and Strengths

The principal weakness related to this study refers to the fact that, to the best of our knowledge, analyzing the available literature (published until 10 May 2021) an extended, comprehensive, and complete NGMSs performance evaluation has not yet been performed and carried out in the literature, although some studies on this topic are noteworthy (e.g., [105,112,113,114,115]). For this reason, a definitive conclusion regarding the use of this technology cannot yet be drawn, even if it is generally recognized that NGMSs are generally less reliable compared to high-end devices [101,102,103]. Due to the keywords defined a priori and then inserted in the search queries, this work mainly investigates two application fields (citizen science and worker health) but other application fields deserve to be taken into consideration in future studies. Another weakness could be related to the inclusion/exclusion criteria chosen a priori: in this review, only the main pollutants related to airborne compartment were considered, but there are also other types of monitors and sensors concerning the real-time evaluation of human health (e.g., smartwatches, wrist band, under-skin sensors) that may be considered in exposure assessment studies. In future studies, it will also be appropriate to find information regarding calibration and the lifetime of each sensor. It will also be necessary to investigate the entire monitoring systems that include the NGMSs, in order to properly integrate these technologies into a total monitoring system, necessary for its operation. In this regard, all the components of the entire system will be evaluated jointly, to favor each integration.

The strengths of this review refer to the fact of having systematically reported the scientific papers on this topic, to facilitate the choice/revision of a sensor/monitor by the scientific community, referring to the more detailed scientific literature, in a fast growing and rapid evolving research field. Moreover, another important strength regards an up-to-date collection of the type of NGMSs used in literature and their applicability. Furthermore, general suggestions were given, regarding the various aspects considered in the review.

## 4. Conclusions

This study presented the state of the art of recent studies based on NGMSs. The reviewing process was conducting systematically with the aim to extrapolate information about the specs, the technologies, and the applicability of NGMSs in both environmental and occupational exposure assessment. Several technologies have emerged concerning the NGMSs used in the literature, but the most relevant ones are based on electrochemical sensing for gas monitoring and light scattering principles for PM monitoring. NGMSs cannot be used as reference instrumentation for regulatory purpose, but at the same time they can be easily adapted to more specific applications, improving exposure assessment studies in terms of spatiotemporal resolution, wearability, and adaptability to different types of projects and applications. If paired with reference methods, NGMSs can elevate the exposure assessment studies to a higher level of detail. Nevertheless, improvements are needed to further enhance the performances of NGMSs and allow their wider use in the field of exposure assessment.

## Figures and Tables

**Table 1:** Query used in the three different databases (Scopus, ISI Web of Knowledge, PubMed).

Database	Search Query
Scopus	(TITLE-ABS-KEY (“air quality” OR pm OR gas * OR air OR “air pollut *” OR pollut *)) AND (TITLE-ABS-KEY (“personal exposure” OR “human exposure” OR exposome)) AND (TITLE-ABS-KEY (“sensor network” OR “wearable sens *” OR “crowd sensing” OR “participatory sensing” OR “mobile sensor node” OR “low cost sensor” OR “citizen science” OR “mobile phone app *” OR “lightweight device *” OR “bluetooth” OR “air pollution sens *” OR “portable device” OR server OR cloud OR “miniaturized sensor *”))
ISI Web of Knowledge	(TS = (“air quality” OR “pm” OR “gas *” OR “air” OR “air pollut *” OR “pollut *”)) AND (TS = (“personal exposure” OR “human exposure” OR “exposome”)) AND (TS = (“sensor network” OR “wearable sens *” OR “crowd sensing” OR “participatory sensing” OR “mobile sensor node” OR “low cost sensor” OR “citizen science” OR “mobile phone app *” OR “lightweight device *” OR “bluetooth” OR “air pollution sens *” OR “portable device” OR server OR cloud OR “miniaturized sensor *”))
PubMed	(((((((personal exposure) OR (human exposure)) OR (exposome))) AND (((((((air quality) OR (pm)) OR (gas *)) OR (air)) OR (air pollut *)) OR (pollut *)))) AND ((((((((((((((((sensor network) OR (wearable sens *)) OR (crowd sensing)) OR (participatory sensing)) OR (mobile sensor node)) OR (low cost sensor)) OR (citizen science)) OR (mobile phone app *)) OR (lightweight device)) OR (bluetooth)) OR (air pollution sens *)) OR (portable device *)) OR (server)) OR (cloud)) OR (miniaturized sensor)))) NOT (pollut *))

**Table 2:** Pollutants and other parameters (temperature—T; relative humidity—RH) investigated, relative NGMSs used (only those available), relative technologies (EC—electrochemical; MOS—metal oxide semiconductor; LS—light scattering; CS—capacitive sensing; Th—thermistor; SBG—silicon band gap; n.a.—not available) and the number of involved papers in which sensors were made explicit and used. Monitors are marked by “*” to distinguish them from the sensors. Technical features of the selected sensors are summarized in Tables S2 and S3 (Supplementary Materials).

Pollutants	Sensor Name/Models	Sensor Technology	Available Papers	References
NO_2_	Alphasense NO2-A1	EC	1	[30]
	Alphasense NO2-A43F	EC	4	[28,29,31,54]
	Alphasense NO2-B43F	EC	5	[25,32,34,35,36]
	e2V MiCS-2710	MOS	2	[55,56]
	* Sailbri Cooper Inc SCI-608	n.a.	1	[57]
	SGX SensorTech MiCS 2714	MOS	1	[58]
	SGX SensorTech MiCS-4514	MOS	3	[27,52,59]
O_3_	Alphasense OX-A431	EC	5	[28,29,31,54,60]
	Alphasense OX-B431	EC	5	[25,34,35,61,62]
	Nissha FIS SP-61	MOS	1	[35]
	* Sailbri Cooper Inc SCI-608	n.a.	1	[57]
	SGX Sensortech MICS 2614	MOS	3	[19,32,58]
	Winsen MQ-131	MOS	1	[63]
CO	Alphasense CO-A4	EC	2	[29,60]
	Alphasense CO-AF	EC	1	[30]
	Alphasense CO-B41	EC	4	[25,34,61,62]
	e2V MiCS-5525	MOS	1	[64]
	Figaro TGS 2442	MOS	1	[58]
	* Sailbri Cooper Inc SCI-608	n.a.	1	[57]
	SGX SensorTech MiCS-4514	MOS	3	[27,52,59]
	Winsen MQ-7	MOS	1	[63]
VOC	Sensirion SGP30	MOS	1	[40]
	Sensirion SGPC3	MOS	1	[19]
PM	Honeywell HPMA115S0	LS	1	[65]
	Nova Fitness SDS-011	LS	1	[36]
	Plantower PMS3003	LS	3	[27,47,66]
	Plantower pms5003	LS	3	[52,67,68]
	Sharp Electronics GP2Y1010AU0F	LS	3	[61,63,69]
	* TSI OPS3330	LS	1	[70]
PM_2.5_	Alphasense OPC-N2	LS	1	[71]
	Plantower pms3003	LS	4	[31,47,72,73]
	* RTI International MicroPEM	LS	1	[74]
	* Sailbri Cooper Inc SCI-608	LS	1	[57]
	Sharp DN7C3CA006	LS	2	[62,75]
	Shinyei PPD42NS	LS	1	[76]
	Shinyei PPD60PV- T2	LS	2	[50,77]
PM_10_	* Sailbri Cooper Inc SCI-608	LS	1	[57]
**Other** **Parameters**				
T–RH	Adafruit AM2302	CS–TH	1	[61]
	Aosong Electronics DHT22	CS-TH	1	[52]
	CMOS sensor (HTU-21D)	CS-TH	1	[54]
	Cozir AH-1	ND	1	[31]
	* Sailbri Cooper Inc SCI-608	ND	1	[57]
	Sensirion SCD30	CS-SBG	1	[36]
	Sensirion SHT15	CS-SBG	2	[47,73]
	Sensirion SHT31	CS-SBG	1	[66]
	Sensirion SHT75	CS-SBG	1	[60]
	SST sensing CO2S-A	ND	1	[34]
	Texas Instruments HDC1080	CS-TH	1	[27]
GC	G.TOP FGPMMOPA6H	GPS	1	[27]
	Adafruit Ultimate GPS chip	GPS	1	[52]

## Supplementary Materials

The following are available online at https://www.mdpi.com/article/10.3390/s21134513/s1: Table S1: Studies object of the review. In this table, all the sources considered in this review are reported, divided by author, title, reference, and the corresponding number of citations in the text. Table S2: Gas sensor and monitor (*) characteristics reported in the papers analyzed in this review. In the case of missing data within the reference papers, data were acquired from the literature cited in the bibliography or from external sources (retailer’s website); n.a.—not available. Table S3: Particulate sensors and monitors (*) characteristics reported in the papers object of this review. In the case of missing data within the reference papers, data were acquired from the literature cited in the bibliography or from external sources (retailer’s website); n.a.—not available.

## Abbreviations

BC	Black Carbon
BLE	Bluetooth Low Energy technology
CO	Carbon monoxide
CO_2_	Carbon dioxide
CS	Capacitive Sensing
DRI	Direct Reading Instrument(s)
EC	Electrochemical (sensor)
GC	Geographic Coordinates
GPS	Global Position System
LCM(s)	Low-Cost Monitor(s)
LS	Light Scattering (sensor)
ME(s)	Micro-Environment(s)
MM(s)	Miniaturized Monitor(s)
ND	Not Defined
NGMS(s)	“Next Generation” Monitors and Sensor(s)
NO	Nitric oxide
NO_2_	Nitrogen dioxide
NO_x_	Nitric oxides
O_3_	Ozone
PM	(Airborne) Particulate Matter
PM_2.5_	Airborne Particulate Matter (PM) with aerodynamic diameter below 2.5 µm
PM_10_	Airborne Particulate Matter (PM) with aerodynamic diameter below 10 µm
QTF	Quartz Tuning Fork (sensor)
RH	Relative Humidity
SBG	Silicon Band Gap (sensor)
SO_2_	Sulfur Dioxide
T	Temperature
Th	Thermistor
VOC	Volatile Organic Compounds
WM(s)	Wearable Monitor(s)
WSN	Wireless Sensor Network
