# Supplementary material for: Features and Practicability of the Next-Generation Sensors and Monitors for Exposure Assessment to Airborne Pollutants: A Systematic Review
Source: Sensors (Basel). 2021 Jun 30;21(13):4513. doi: 10.3390/s21134513 (PMC8271362; doi:10.3390/s21134513)
Supplement: Supplementary file 1 [file sensors-21-04513-s001.zip › sensors-1247283-supplementary.pdf]

sensors

MDPI

Review

Supplementary Materials: Features and Practicability of the Next-Generation Sensors and Monitors for Exposure Assessment to Airborne Pollutants: A Systematic Review

Giacomo Fanti, Francesca Borghi, Andrea Spinazzè, Sabrina Rovelli, Davide Campagnolo, Marta Keller, Andrea Cattaneo, Emanuele Cauda and Domenico Maria Cavallo.

Table S1. Studies object of the review. In this table are reported all the sources considered in this review, divided by author, title, reference and the corresponding number of citations in the text.

First Author	Title	Journal	Year of publication	Citation
Q. Dong	A Cloud-connected NO2 and Ozone Sensor System for Personalized Pediatric Asthma Research and Management	IEEE Sensors Journal	2020	[54]
S. De Vito	A crowdfunded personal air quality monitor infrastructure for active life applications	IEEE International Workshop on Measurement and Networking (M&N)	2017	[60]
T. Becnel	A Distributed Low-Cost Pollution Monitoring Platform	Internet Of Things Journal, Vol. XX, No. XX,	2018	[27]
M. Gao	A distributed network of low-cost continuous reading sensors to measure spatiotemporal variations of PM2.5 in Xi'an, China	Environmental Pollution	2015	[76]
N. Zimmerman	A machine learning calibration model using random forests to improve sensor performance for lower-cost air quality monitoring	Atmospheric Measurement Techniques	2018	[34]
Z. Du	A Miniaturized Particulate Matter Sensing Platform based on CMOS imager and Real Time Image Processing	IEEE Sensors Journal	2018	[41]
W. Yi	A Modular Plug-ANA-Play Sensor System for Urban Air Pollution Monitoring: Design, Implementation and Evaluation	Sensors- MDPI	2017	[37]
Y. Deng	A Novel Wireless Wearable Volatile Organic Compound (VOC) Monitoring Device with Disposable Sensors	Sensors- MDPI	2016	[79]
M. Balanescu	A study on data accuracy for IoT measurements of PMs concentration	International Conference on Control Systems and Computer Science (CSCS)	2019	[43]
B. Dessimond	Academically Produced Air Pollution Sensors for Personal Exposure Assessment: The Canarin Project	Sensors- MDPI	2021	[40]
M. Ueberham	Advanced Smartphone-Based Sensing with Open-Source Task Automation	Sensors- MDPI	2018	[80]
N. Alhakbani	Air Pollution Measures in Riyadh City and Personal Exposure Level	International Conference on Cloud Computing (ICCC)	2015	[81]
M. Balanescu	An Algorithm to Improve Data Accuracy of PMs Concentration Measured with IoT Devices	Advances in Science, Technology and Engineering Systems Journal Vol. 5, No. 2, 180-187	2020	[42]

R. Huang	Application and Evaluation of a Low-cost PM Sensor and Data Fusion with CMAQ Simulations to Quantify the Impacts of Prescribed Burning on Air Quality in Southwestern Georgia, USA	Journal of the Air & Waste Management Association	2021	[47]
J. M. Barcelo-Ordinas	Calibrating Low-Cost Air Quality Sensors Using Multiple Arrays of Sensors	IEEE Wireless Communications and Networking Conference	2018	[32]
N. Castell	Can commercial low-cost sensor platforms contribute to air quality monitoring and exposure estimates?	Environment International	2016	[28]
L. Chatzidiakou	Characterising low-cost sensors in highly portable platforms to quantify personal exposure in diverse environments	Atmospheric Measurement Technique	2019	[29]
J. J. Huck	Combining physiological, environmental and locational sensors for citizen-oriented health applications	Environ Monit Assess	2017	[56]
P. Dutta	Common Sense - Participatory Urban Sensing using a Network of Handheld Air Quality Monitors	Proceedings of the 7th ACM Conference on Embedded Networked Sensor Systems	2009	[94~~5~~]
T. Zhang	Comparison of PM2.5 Exposure in Hazy and Non-Hazy Days in Nanjing, China	Aerosol Air Qual Res.	2017	[74]
S.C.C. Lung	Concurrent Assessment of Personal, Indoor, and Outdoor PM2.5 and PM1 Levels and Source Contributions using Novel Low-cost Sensing Devices	Indoor Air	2020	[66]
R. R. Fletcher	Design and Clinical Feasibility of Personal Wearable Monitor for Measurement of Activity and Environmental Exposure	36th Annual International Conference of the IEEE Engineering in Medicine and Biology Society	2014	[38]
D. Suriano	Design and Development of a Flexible, Plug-and-Play, Cost- Effective Tool for on-Field Evaluation of Gas Sensors	Journal of Sensors	2020	[35]
J. Tryner	Design and evaluation of a portable PM2.5 monitor featuring a low-cost sensor in line with an active filter sampler	Environ. Sci.: Processes Impacts	2019	[67]
K. R. Mallires	Developing a Low-Cost Wearable Personal Exposure Monitor for Studying Respiratory Diseases Using Metal–Oxide Sensors	IEEE SENSORS JOURNAL	2019	[19]
C. Zuidema	Estimating personal exposures from a multi-hazard sensor network	Journal of Exposure Science & Environmental Epidemiology	2019	[61]
S. Mahajan	Evaluation of low-cost sensors for quantitative personal exposure monitoring	Sustainable Cities and Society - ELSEVIER	2020	[68]
Y. Zou	Examining the functional range of commercially available low- cost airborne particle sensors and consequences for monitoring of indoor air quality in residences	John Wiley & Sons Ltd	2019	[65]
Q. Zhang	Flexible gas sensor based on graphene/ethyl cellulose nanocomposite with ultra-low strain response for volatile organic compounds rapid detection	IOP Publishing - Nanotechnology	2018	[3]
C. Lin	High Performance Colorimetric Carbon Monoxide Sensor for Continuous Personal Exposure Monitoring	ACS Sensors	2018	[45]
R. Chew	Identification of Bicycling Periods Using the MicroPEM Personal Exposure Monitor	sensors- MDPI	2019	[53]
T.T. Win-Shwe	Improvement of GPS-attached Pocket PM2.5 Measuring Device for Personal Exposure Assessment	J. UOEH	2020	[91~~2~~]
D. B. Topalović	In search of an optimal in-field calibration method of low-cost gas sensors for ambient air pollutants: Comparison of linear, multilinear and artificial neural network approaches	Atmospheric Environment	2019	[25]
S. Hegde	Indoor Household Particulate Matter Measurements Using a Network of Low-cost Sensors	Aerosol and Air Quality Research	2020	[52]
J. Núñez	Inline Infrared Chemical Identification of Particulate Matter	Sensors- MDPI	2020	[70]
D. Sinaga	Investigation on daily exposure to PM2.5 in Bandung city, Indonesia using low-cost sensor	Journal of Exposure Science & Environmental Epidemiology	2020	[72]
M. Mazaheri	Investigations into factors affecting personal exposure to particles in urban microenvironments using low-cost sensors	Environment International	2018	[77]

F. M. J. Bulot	Long-term field comparison of multiple low-cost particulate matter sensors in an outdoor urban environment	Nature/Scientific Reports	2019	[71]
G. R. McKercher	Low-cost mobile air pollution monitoring in urban environments: a pilot study in Lubbock, Texas.	Environmental Technology	2018	[59]
G. W. Thomas	Low-Cost, Distributed Environmental Monitors for Factory Worker Health	Sensors- MDPI	2018	[62]
M. Magno	Low-Power Gas Sensing using Single Walled Carbon Nano tubes in Wearable Devices	Environmental Sociology	2017	[55]
R. Milton	Mapping Carbon Monoxide Using GPS Tracked Sensors	Environ Monitoring and Assessment-Springer	2007	[901]
X. Qin	Micro Quartz Tuning Fork based PM2.5 Sensor for Personal Exposure Monitoring	IEEE Sensors Journal	2019	[82]
S. Nagendra	Mobile monitoring of air pollution using low cost sensors to visualize spatio-temporal variation of pollutants at urban hotspots	Sustainable Cities and Society	2018	[58]
M. Nyarku	Mobile phones as monitors of personal exposure to air pollution: Is this the future?	PLOS ONE	2018	[1113]
L. B. Frederickson	Monitoring Excess Exposure to Air Pollution for Professional Drivers in London Using Low-Cost Sensors	Atmosphere	2020	[36]
T.H. Wen	Monitoring Street-Level Spatial-Temporal Variations of Carbon Monoxide in Urban Settings Using a Wireless Sensor Network (WSN) Framework	Environmental Research and Public Health	2013	[64]
M. Reid	NaDos: A real-time, wearable, personal exposure monitor for hazardous organic vapors	Sensors and Actuators B: Chemical	2017	[1001]
S. E. West	Particulate matter pollution in an informal settlement in Nairobi: Using citizen science to make the invisible visible	Applied Geography	2020	[923]
L. Yang	People-centric Cognitive Internet of Things for the Quantitative Analysis of Environmental Exposure	IEEE Internet of Things Journal	2017	[889]
H. Agrawaal	Personal Exposure Estimates via Portable and Wireless Sensing and Reporting of Particulate Pollution	Environ. Res. Public Health	2020	[69]
P. W. Oluwasanya	Portable Multi-Sensor Air Quality Monitoring Platform for Personal Exposure Studies	IEEE Instrumentation & Measurement Magazine	2019	[83]
T. Cao	Portable, Ambient PM2.5 Sensor for Human and/Or Animal Exposure Studies	Analytical Letters	2016	[75]
F. Borghi	Precision and Accuracy of a Direct-Reading Miniaturized Monitor in PM2.5 Exposure Assessment	Sensors- MDPI	2018	[50]
R. Wang	Real-Time Ozone Detection Based on a Microfabricated Quartz Crystal Tuning Fork Sensor	Sensors- MDPI	2009	[1045]
L.-W. A. Chen	Schoolchildren's exposure to PM2.5: a student club–based air quality monitoring campaign using low-cost sensors	Air Quality, Atmosphere & Health	2020	[57]
D. K. Arvind	The AirSpeck family of static and mobile wireless air quality monitors	Euromicro Conference on Digital System Design	2016	[84]
J. Liao	The use of bluetooth low energy Beacon systems to estimate indirect personal exposure to household air pollution	Journal of Exposure Science & Environmental Epidemiology	2019	[78]
M.I. Mead	The use of electrochemical sensors for monitoring urban air quality in low-cost, high-density networks	Atmospheric Environment	2012	[30]
Y. Deng	Unraveling fabrication and calibration of wearable gas monitor for use under free-living conditions	Annual International Conference of the IEEE Engineering in Medicine and Biology Society (EMBC)	2016	[51]
L. Chatzidiakou	Using low-cost sensor technologies and advanced computational methods to improve dose estimations in health panel studies: results of the AIRLESS project	Journal of Exposure Science & Environmental Epidemiology	2020	[33]
M. Liu	Using low-cost sensors to monitor indoor, outdoor, and personal ozone concentrations in Beijing, China	Environmental Science, Processes & Impacts	2020	[31]
K. K. Barkjohn	Using Low-cost Sensors to Quantify the Effects of Air Filtration on Indoor and Personal Exposure Relevant PM2.5 Concentrations in Beijing, China	Aerosol and Air Quality Research	2020	[73]

M. J. Nieuwenhuijsen	Variability in and Agreement between Modeled and Personal Continuously Measured Black Carbon Levels Using Novel Smartphone and Sensor Technologies	Environmental Science & Technology	2015	[39]
N. Dam	Wearable Sensors for Analyzing Personal Exposure to Air Pollution	IEE - SIEDS	2017	[63]
M. Ueberham	Wearable sensors for multifactorial personal exposure measurements – A ranking study	Environment International	2018	[85]
J. E. Johnston	Youth Engaged Participatory Air Monitoring: A 'Day in the Life' in Urban Environmental Justice Communities	Environmental Research and Public Health	2019	[86]

Table S2. Gas sensors and monitors (*) characteristics reported in the papers analyzed in this review. In the case of missing data within the reference papers, data were acquired from the literature cited in the bibliography or from external sources (retailer's website). n.a.: not available.

Gas sensors and monitors						
**Pollutants**	**Sensor name/models**	**Sensor Technology**	**Dimension**	**Weight**	**Operational Range**	**Sensitivity**
NO_2	Alphasense NO2-A1	Electrochemical	20,2x16,5 mm	<6g	0-20 ppm	-400 to -750 nA/ppm at 10ppm NO_2
	Alphasense NO2-A43F	Electrochemical	20,2x16,5 mm	<6g	0-20 ppm	-175 to -450 nA/ppm at 2ppm NO_2
	Alphasense NO2-B43F	Electrochemical	32,3x16,5 mm	<13g	0-20 ppm	-200 to -650 nA/ppm at 2ppm NO_2
	e2V MiCS-2710	Metal Oxide semiconductor	9,15x13,9 mm	n.a.	0-5 ppm	n.a.
	SGX SensorTech MiCS 2714	Metal Oxide semiconductor	5x7x1,55 mm	4g	0-10 ppm	n.a.
	SGX SensorTech MiCS-4514	Metal Oxide semiconductor	5x7x1,55 mm	4,087g	0-10 ppm	n.a.
O_3	Alphasense OX-A431	Electrochemical	20,2x16,5 mm	<6g	0-50 ppm	-200 to -650 nA/ppm at 1ppm O_3
	Alphasense Ox-B431	Electrochemical	32,3x16,5 mm	<6g	0-50 ppm	-225 to -750 nA/ppm at 1ppm O_3
	Nissha FIS SP-61	Metal Oxide semiconductor	51x37x22 mm	15g	0-250 ppb	n.a.
	SGX Sensortech MICS 2614	Metal Oxide semiconductor	5x7x1,55 mm	4g	10-1000 ppb	n.a.
	Winsen MQ-131	Metal Oxide semiconductor	24x19 mm	4,5g	10-1000 ppb	n.a.
CO	Alphasense CO-A4	Electrochemical	20,2x16,5 mm	<6g	0-500 ppm	220 to 410 nA/ppm at 2ppm CO
	Alphasense CO-AF	Electrochemical	20,2x16,5 mm	<6g	0-5000 ppm	55 to 90 nA/ppm at 400ppm CO
	Alphasense CO-B4	Electrochemical	32,3x16,5 mm	<13g	0-1000 ppm	420 to 650 nA/ppm at 2ppm CO
	e2V MiCS-5525	Metal Oxide semiconductor	11x24,5 mm	n.a.	0-1000 ppm	n.a.
	Figaro TGS 2442	Metal Oxide semiconductor	9,2x22,7 mm	n.a.	30-1000 ppm	n.a.
	SGX SensorTech MiCS-4514	Metal Oxide semiconductor	5x7x1,55 mm	4,087g	1-1000 ppm	n.a.
	Winsen MQ-7	Metal Oxide semiconductor	16,5x14,54 mm	n.a.	10-500 ppm	n.a.
VOC	Sensirion SGP30	Metal Oxide semiconductor	2,45x2,45x0,9 mm	n.a.	0-60000 ppb	n.a.
	Sensirion SGPC3		2,45x2,45x0,9 mm	n.a.	0-60000 ppb	n.a.
*Sailbri Cooper Inc SCI-608 can monitor NO_2, O_3, CO; Dimension: 220x200x300 mm; Weight: 2,6kg; Operational Range: 0-500 ppm						

Table S3. Particulate sensors and monitors (*) characteristics reported in the papers object of this review. In the case of missing data within the reference papers, data were acquired from the literature cited in the bibliography or from external sources (retailer's website). n.a.: not available.

PM sensors and monitors						
**Pollutants**	**Sensor name/models**	**Sensor Technology**	**Dimension**	**Weight**	**Operational Range**	**Sensitivity**
PM	Honeywell HPMA115S0	Light Scattering	44x36x12mm	82,330g	0-1000 μg/m³	15 μg/m³
	Nova Fitness SDS-011		71x70x23 mm	n.a.	0-999,9 μg/m³	n.a.
	Plantower PMS3003		65x42x23 mm	35g	0-500 μg/m³	1 μg/m³
	Plantower pms5003		50x38x21 mm	42g	0-500 μg/m³	1 μg/m³
	Sharp Electronics GP2Y1010AU0F		46x34x18,3 mm	16g	0-600 μg/m³	0,1 μg/m³
$PM_{2.5}$	Alphasense OPC-N2	Light Scattering	75x60x63 mm	105g	0-1500 μg/m³	0,1 μg/m³
	Plantower pms3003		65x42x23 mm	35g	0-500 μg/m³	1 μg/m³
	*RTI International MicroPEM		n.a.	240g	0-10000 μg/m³	5 μg/m³
	Sharp DN7C3CA006		51x53x40 mm	n.a.	25-500 μg/m³	0,1 μg/m³
	Shinyei PPD42NS		59x45x22 mm	28 g	0-500 μg/m³	9,1 μg/m³
	Shinyei PPD60PV- T2		88x60x22 mm	36 g	0-500 μg/m³	29 μg/m³

*Sailbri Cooper Inc SCI-608 can monitor $PM_{2.5}$ and PM_{10}; Sensor Technology is Light Scattering; Dimension: 220x200x300 mm; Weight: 2,6kg; Operational Range:0-1000 μg/m³; Sensitivity: 5 μg/m³

*TSI OPS3330 can monitor several PM size fractions; Sensor Technology is Light Scattering; Dimension: 135x216x224 mm; Weight: 2,0kg; Operational Range: 0,001-275 μg/m³; Sensitivity: 0,001 μg/m³
